# Extragastrointestinal stromal tumor of the mesoappendix: CT findings and a review of the literature

**DOI:** 10.1186/1477-7819-10-211

**Published:** 2012-10-07

**Authors:** Wenhua Li, Yanfen Cui, Gang Ren, Jun Wang, Xiangru Wu

**Affiliations:** 1 Department of Radiology, Xinhua Hospital affiliated to Shanghai Jiao Tong University School of Medicine, 1665 Kong Jiang Road, Shanghai 200092, China; 2 Department of Pediatric Surgery, Xinhua Hospital affiliated to Shanghai Jiao Tong University School of Medicine, 1665 Kong Jiang Road, Shanghai, 200092, China; 3 Department of Pathology, Xinhua Hospital affiliated to Shanghai Jiao Tong University School of Medicine, 1665 Kong Jiang Road, Shanghai, 200092, China

**Keywords:** Children, CT, Extragastrointestinal stromal tumor

## Abstract

**Background:**

Gastrointestinal stromal tumors (GISTs) are nonepithelial, mesenchymal neoplasms that rarely occur in children.

**Case presentation:**

We present a unique case of a GIST that developed outside the gastrointestinal tract within the mesoappendix of a 6-year old boy. Computed tomography (CT) revealed a slightly lobulated, homogeneous soft-tissue mass, with marked contrast enhancement.

**Conclusion:**

This case study provides new insight into the CT appearance of extragastrointestinal stromal tumors.

## Background

Gastrointestinal stromal tumors (GISTs) are nonepithelial, mesenchymal tumors that arise from the intestinal cells of Cajal (ICC) or their stem cell precursors, and constitute approximately 2% of all neoplasms of the gastrointestinal tract [1]. These tumors very rarely occur in children and young adults, who account for approximately 1.4% of all patients with GISTs [2]. The tumors arise in the stomach in 40% to 70% of cases and in the small intestine in 20% to 40% of cases, while less than 10% of tumors occur in the esophagus, colon, or rectum; they usually present in adults over 40 years of age, with a peak incidence in the sixth and seventh decades [3]. They may also originate from extragastrointestinal tract sites, such as the omentum, mesentery, retroperitoneum, pancreas, fallopian tubes, or uterus [4,5]. These are termed extragastrointestinal stromal tumors (eGISTs), and usually behave more aggressively. In this report, we present the case of a GIST of the mesoappendix, and focus on the CT findings and a review of the literature.

## Case presentation

**Figure 1:**
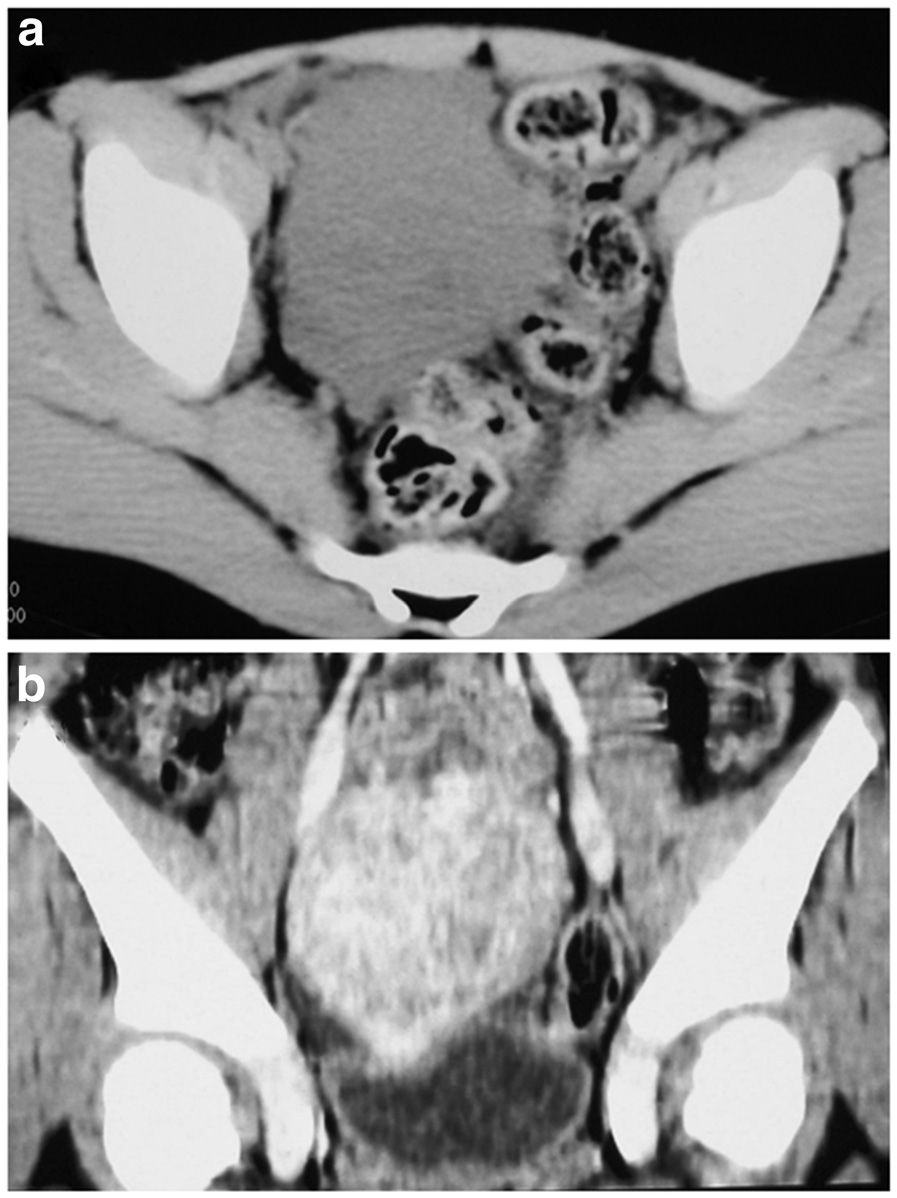
**CT of the pelvis (a): Axial pre-contrast image shows a well-defined, slightly lobulated homogeneous soft-tissue mass in the right lower abdominal cavity.** (**b**): Reconstructed coronal image shows the tumor with homogeneous enhancement without necrotic or cystic components.

**Figure 2:**
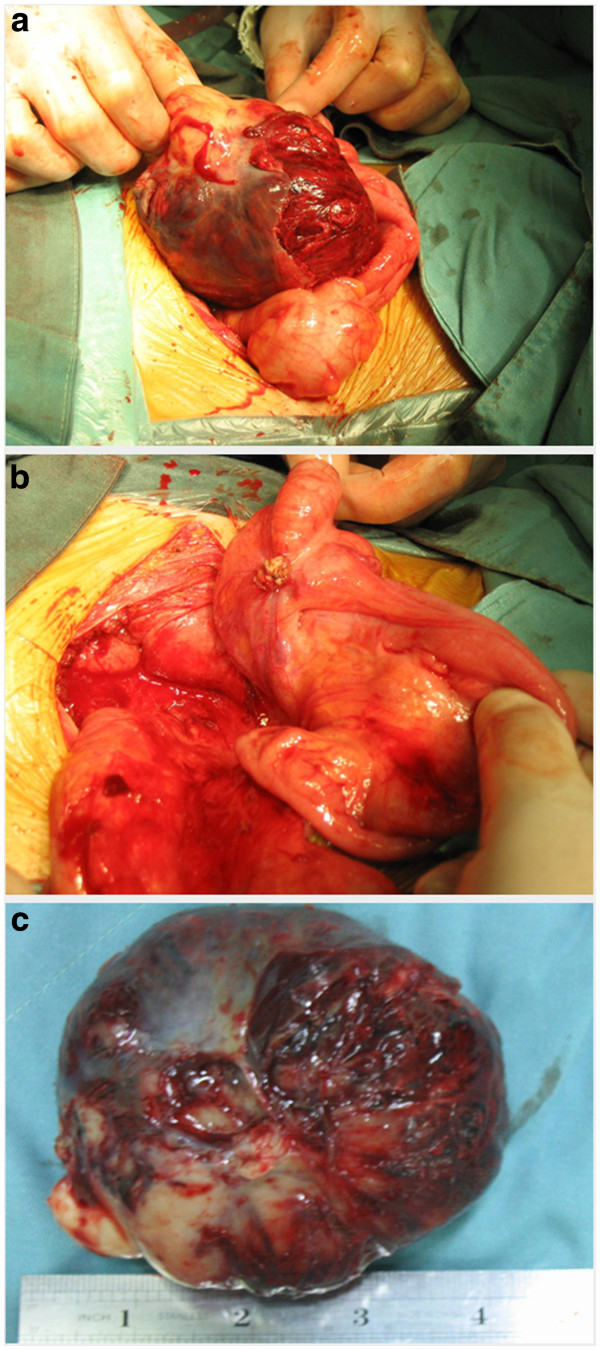
**Operative image (a): The tan-colored tumor arises from the mesoappendix.** (**b**): The tumor was completely removed without adhesions to the appendix or other organs or structures. (**c**): Gross findings of the resected tumor. The tumor is tan-colored with an incomplete capsule.

**Figure 3:**
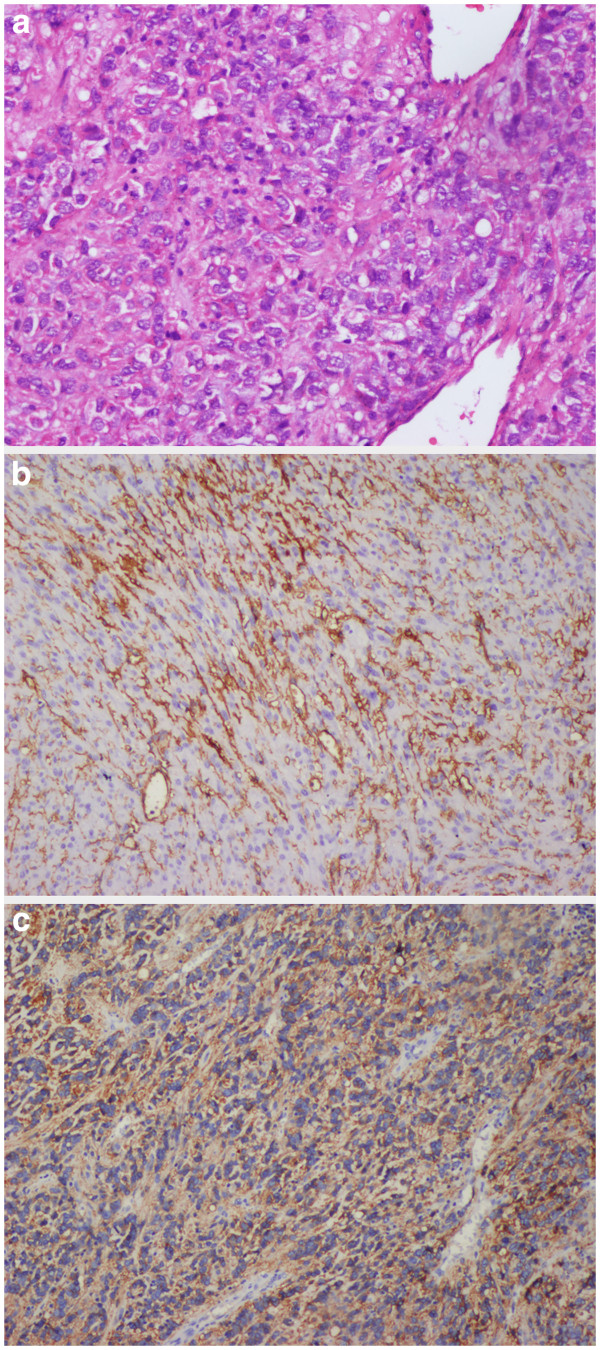
**High-power photomicrograph (hematoxylin-eosin, ×200) (a): The tumor is composed of spindle and epithelioid cells.** (**b**): CD117 immunostaining of the tumor; CD117 is diffusely and strongly positive in the tumor cells. (**c**): CD34 immunostaining of the tumor; D34 is diffusely and strongly positive in the tumor cells.

A developmentally normal 6-year-old boy was admitted to our hospital for evaluation of lower abdominal pain on the right side. Physical examination revealed a hard and well-demarcated mass. Routine laboratory tests were normal. Subsequently, an abdominal CT was performed using a GE LightSpeed scanner (GE Medical Systems, Milwaukee, WI, USA), with parameters of 120 kV and 180 mA. This revealed a mildly lobulated, well-defined homogeneous soft-tissue mass, 5.0 × 5.7 × 6.7 cm in size, in the right lower abdominal cavity, and there was no evidence of pelvic lymphadenopathy (Figure 1). The lesion had a density of 41 HU (Hounsfield units); it had no hemorrhagic, necrotic, or cystic components. Following infusion of a contrast agent, the tumor demonstrated marked homogeneous enhancement, and a density of 81 HU. Laparotomy revealed a tan-colored mass arising from the mesoappendix, without adhesions to the appendix or other organs and structures (Figure 2). Macroscopically, the mass was well-circumscribed, with an incomplete capsule, and all margins were negative. Microscopically, the tumor consisted of proliferating spindle cells and epithelioid cells. Mitotic figures were noted in 12 of 50 high-power fields. Immunohistochemical staining revealed that the tumor was diffusely and strongly positive for c-Kit (CD117) (Figure 3), myeloid stem cell antigen (CD34), DOG1, and Ki-67, slightly positive for vimentin, and negative for smooth muscle actin (SMA), neuron-specific enolase (NSE), S-100, and desmin. Based on these morphological and immunohistochemical findings, the final pathological diagnosis was that of a malignant gastrointestinal stromal tumor of the mesoappendix. The patient was treated by administration of Glevec as an adjuvant postoperative chemotherapy and has been living disease-free for 9 months of follow-up.

## Discussion

Primary eGISTs are distinctly uncommon, according to previous reports [4,5]. An extensive literature review was undertaken, using the key words ‘GIST,’ ‘Cajal cells,’ ‘KIT,’ and ‘Imatinib.’ To the best of our knowledge, there have been no previous reports of a GIST arising from the mesoappendix. This case was positive for CD34 and CD117, which supports the diagnosis, as this test distinguishes a GIST from mesenchymal tumors arising from smooth muscle cells, such as leiomyomas, leiomyoblastomas, and leiomyosarcomas. The origin of GISTs was at first attributed to the ICC, but it is now recognized that they arise from multipotential mesenchymal stem cells [6]. ICC or ICC-like cells have also been described in various organs, excluding the gastrointestinal tract [7]. In the case we present, the tumor may have originated from ICC or ICC-like cells and multipotential mesenchymal stem cells in the mesoappendix.

It can be difficult to confirm a diagnosis of GIST, and specific morphologic, immunohistochemical, and molecular analyses are required. The majority of GISTs have a uniform appearance that falls into one of three categories: spindle cell, epitheloid cell, and mixed cell. On immunohistochemistry, 90% to 95% of GISTs are diffusely and strongly positive for CD117 (c-Kit), but this is no longer considered an absolute criterion. Not all CD117-positive tumors are GISTs, as melanomas, synovial sarcomas, desmoid tumors, and schwannomas can also be positive for this marker [1]. Some CD117 negative GISTs have an epithelioid morphology and arise in the stomach or outside the gut. Therefore, CD34 has been proposed as a more reproducible marker, and is positive in 80% to 85% of cases. The spindle cell type is more likely to stain with CD34 than the epithelioid type, and the mixed spindle-epithelioid type is more likely to stain with CD34 than the nonmixed type. Smooth muscle actin is expressed in 30% to 40% of cases. S-100 positivity is present in up to 5% of these tumors, but is relatively more common in small intestinal GISTs. In general, GISTs tend to be negative for desmin, or weakly positive in 1% to 2% of cases [1].

The spectrum of the clinical presentation of GISTs is broad and depends on tumor location and size, with approximately 70% of patients developing such symptoms as abdominal pain, gastrointestinal bleeding, and mass effects. The remaining 20% to 30% present incidentally during radiological imaging or surgery for some other cause. The tumors occur with equal frequency in both sexes, although some studies have shown a male preponderance [1-3].

Since the classification of GIST as a distinct entity, there has been an increased interest in defining the imaging characteristics. The tumors usually commence in the bowel wall, but may extend to involve either the mucosal or the serosal surfaces. Tumor size ranges from 1 cm to 35 cm, with a median of 5 cm. The tumor margins are well-defined in approximately two-thirds of cases. They can be of any size, but large tumors, in particular, can have areas of hemorrhage and necrosis that demonstrate a heterogeneous appearance on imaging. The enhancement pattern can vary from homogeneous to heterogeneous [8-10]. According to the World Health Organization criteria [11], malignant potential is assessed by tumor size, mitotic count, and the cell proliferative index. Tumors smaller than 5 cm in diameter are usually benign, those between 5 and 10 cm are of uncertain malignant potential, and those larger than 10 cm are usually malignant. This case was a low-grade malignant GIST according to these criteria, and to the criteria of a previous study [8-11].

## Conclusions

In summary, we present the case of a GIST without hemorrhagic, necrotic, or cystic components on CT. Radiologists need to be aware of the imaging characteristics of eGISTs. This rare case provides new insight into the CT presentation of eGISTs.

## Consent

Written informed consent was obtained from the patient for publication of this case report and any accompanying images. A copy of the written consent is available for review by the editor-in-chief of this journal.

## Abbreviations

CT: computed tomography; eGIST: extragastrointestinal stromal tumor; GIST: gastrointestinal stromal tumor; HU: Hounsfield unit; ICC: intestinal cells of Cajal; NSE: neuron-specific enolase; SMA: smooth muscle actin.

## Competing interests

The authors’ declare that they have no competing interests.

## Authors’ contributions

WH Li, YF Cui, and G Ren contributed as diagnostic radiologists. J Wang contributed as pediatric surgeon and performed the operation. XR Wu contributed as a pathologist. All authors read and approved the final manuscript.
